# Variability in Intravenous Insulin Infusion Management in Italian Intensive Care Units: A National Cross‐Sectional Survey of Reported Nursing Practice

**DOI:** 10.1111/nicc.70656

**Published:** 2026-08-31

**Authors:** Carmelo Pujia, Giuseppe Neri, Helenia Mastrangelo, Giuseppe Mazza, Eugenio Biamonte, Caterina Mercuri, Vincenzo Bosco, Tiziana Montalcini, Eugenio Garofalo

**Affiliations:** ^1^ Department of Medical and Surgical Sciences Magna Graecia University Catanzaro Italy; ^2^ ASP Catanzaro Catanzaro Italy; ^3^ Department of Clinical and Experimental Medicine University “Magna Graecia” of Catanzaro Catanzaro Italy

**Keywords:** glucose monitoring, glycaemic control, hyperglycaemia, insulin infusion, intensive care units, nursing practice, practice variability

## Abstract

**Background:**

Hyperglycaemia is common in critically ill patients and is associated with increased morbidity and mortality. Continuous intravenous insulin infusion is widely used for glycaemic control in intensive care units; however, its implementation is complex and requires frequent monitoring and substantial nursing involvement.

**Aims:**

To describe reported practices in continuous intravenous insulin infusion management across Italian intensive care units and to explore variability in glycaemic targets, insulin initiation thresholds, glucose measurement methods and monitoring frequency according to healthcare setting.

**Study Design:**

A national cross‐sectional survey. A structured questionnaire was distributed to intensive care unit nurses and nurse coordinators in Italy between September and November 2025. Data were collected on institutional characteristics, insulin infusion protocols, glycaemic targets, initiation thresholds, glucose measurement methods and monitoring practices. Exploratory comparisons by healthcare setting used cross‐tabulations and exact tests, as appropriate.

**Results:**

A total of 128 valid responses were analysed. Standardised insulin infusion protocols were reported by 119 respondents (93.0%), but considerable variability was observed in reported practice. The most commonly reported insulin initiation threshold was 180 mg/dL (50.0%). Exploratory comparisons suggested differences across healthcare settings in initiation thresholds, glucose measurement methods and monitoring frequency before glycaemic stabilisation. The median reported monitoring interval before stabilisation was 120 min (interquartile range 60–240), exceeding the recommended hourly frequency during glycaemic instability.

**Conclusions:**

Despite widespread reported protocol availability, substantial variability remained in reported continuous intravenous insulin infusion management, particularly in initiation thresholds, glucose measurement methods and monitoring before glycaemic stabilisation. Because workload, staffing and protocol adherence were not directly measured, they should be considered possible explanations rather than study findings.

**Relevance to Clinical Practice:**

The findings support review of local insulin infusion protocols, explicit monitoring intervals during glycaemic instability, staff education, audit and feedback and escalation processes when glucose checks are delayed.

## Impact Statements

- What is known about the topic
  ○ Hyperglycaemia is common in critically ill patients and is associated with adverse clinical outcomes.
  ○ Continuous intravenous insulin infusion is the standard approach for glycaemic control but requires intensive monitoring and nursing input.
  ○ Adherence to guideline‐recommended monitoring and treatment strategies is often challenging in routine clinical practice.
- What this paper adds
  ○ Reported continuous intravenous insulin infusion management practices vary across healthcare settings despite high reported availability of standardised protocols.
  ○ The greatest variability concerns insulin initiation thresholds, glucose measurement methods and glucose‐monitoring frequency before glycaemic stabilisation.
  ○ Reported monitoring intervals before glycaemic stabilisation often exceeded the frequency recommended during glycaemic instability, suggesting a clinically relevant implementation gap in routine intensive care nursing practice.

**Abbreviations**
CGM: continuous glucose monitoring
CII: continuous intravenous insulin infusion
ICU: intensive care unit
SCCM: Society of Critical Care Medicine

## 1 Introduction

Hyperglycaemia is frequently observed in critically ill patients, affecting both individuals with pre‐existing diabetes and those who develop stress‐induced metabolic disturbances during acute illness [1, 2]. Elevated blood glucose levels have consistently been associated with increased complication rates, prolonged hospitalisation and higher mortality in intensive care settings [1, 2, 3]. In particular, postoperative cardiac surgery patients and individuals with severe infections represent high‐risk populations in whom glycaemic dysregulation may significantly influence clinical outcomes [2, 3].

Continuous intravenous insulin infusion (CII) is widely regarded as the standard strategy for managing hyperglycaemia in the ICU because of its rapid onset of action and titratability in response to rapidly changing metabolic conditions [1, 4]. However, its implementation in routine clinical practice is complex and resource intensive. Safe and effective CII management requires frequent glucose monitoring, timely dose adjustments and strict adherence to treatment protocols, with substantial nursing involvement [5, 6].

Accurate glucose measurement is a key prerequisite for safe insulin titration. In critically ill patients, capillary and venous glucose measurements may show reduced reliability, particularly in the presence of haemodynamic instability, potentially leading to clinically relevant dosing errors [7]. These limitations may contribute to variability in bedside practice and increase the risk of both hypoglycaemia and uncontrolled hyperglycaemia [1].

Emerging technologies, including continuous glucose monitoring (CGM) and closed‐loop insulin delivery systems, have shown promising results in improving glycaemic control while reducing glucose variability and hypoglycaemia risk [8, 9, 10, 11]. During the COVID‐19 pandemic, CGM was also used to reduce healthcare worker exposure and optimise resource utilisation [9]. Nevertheless, despite growing evidence supporting technological integration, adoption remains heterogeneous across institutions [11, 12].

Recent literature suggests that nurse‐led insulin infusion protocols may achieve glycaemic control comparable to physician‐managed approaches without increasing adverse outcomes [5, 6]. These findings reinforce the importance of structured protocol implementation, staff training and organisational support in daily ICU practice.

Recent Society of Critical Care Medicine (SCCM) guidelines have provided updated recommendations for glycaemic control in critically ill adults. Specifically, persistent hyperglycaemia ≥ 180 mg/dL should trigger glycaemic management protocols and procedures (Good Practice Statement) [13]. In addition, the guideline suggests against titrating insulin infusion to intensive glucose targets of 80–139 mg/dL, favouring a more conventional range of 140–200 mg/dL to reduce hypoglycaemia (conditional recommendation, moderate certainty of evidence). For the acute management of hyperglycaemia, the guideline also suggests continuous intravenous insulin infusion rather than intermittent subcutaneous insulin (conditional recommendation, very low certainty), frequent glucose monitoring during periods of glycaemic instability (≤ 1 h) (conditional recommendation, low certainty), and the use of protocols incorporating explicit clinical decision‐support tools for insulin titration (conditional recommendation, moderate certainty) [13].

However, adherence to the recommended monitoring intensity appears challenging in routine ICU practice. In a pilot study conducted in the United States among critically ill adults receiving intravenous insulin infusion, Lal et al. reported that compliance with hourly blood glucose monitoring was only 12.6%, with 57% of 1411 glucose measurements performed at intervals of 91–179 min rather than within the expected hourly timeframe [14]. Importantly, delayed monitoring may have clinically relevant consequences. In another United States intensive care unit study, Garg et al. found that, during an insulin infusion protocol in the ICU, 36.1% of glucose measurements were obtained more than 10 min after the recommended time, and delayed monitoring was associated with a significantly higher proportion of blood glucose values < 80 mg/dL compared with on‐time monitoring (17.8% vs. 11.6%, *p* < 0.001) [15]. These findings suggest that maintaining guideline‐recommended monitoring intervals at the bedside may be difficult in real‐world critical care settings, where repeated point‐of‐care glucose testing and frequent insulin dose adjustments are labour‐intensive and must be integrated into broader ICU workflows [14, 15].

Nevertheless, the extent to which these recommendations are translated into routine ICU practice remains uncertain. Differences in glycaemic targets, insulin initiation thresholds, monitoring strategies and protocol organisation may reflect local resources, staffing levels, governance models and clinical culture. Therefore, this national cross‐sectional survey aimed to describe reported practices in continuous intravenous insulin infusion management among ICU nurses and nurse coordinators in Italy, focusing on protocol adoption, glycaemic targets, insulin initiation thresholds, glucose measurement methods and monitoring strategies. In addition, the study explored whether these reported practices varied according to healthcare setting and considered their alignment with current guideline recommendations [13].

## 2 Methods

### 2.1 Study Design

This study was a national cross‐sectional survey designed to investigate self‐reported practices related to the management of continuous intravenous insulin infusion (CII) among ICU nurses and nurse coordinators in Italy.

### 2.2 Setting and Participants

For this study, nurse coordinators were registered nurses with formal organisational or managerial responsibility within an intensive care unit, such as unit coordinators or charge nurses. Data were collected between September and November 2025 among ICU nurses and nurse coordinators working in Italy. Participants were recruited using convenience and snowball sampling strategies through professional networks and institutional contacts. Eligible respondents were nurses or nurse coordinators working in ICUs and directly involved in, or familiar with, the bedside management of continuous intravenous insulin infusion.

The unit of analysis was the individual respondent. Responses were interpreted as self‐reported descriptions of usual practice or perceived unit practice, rather than as audited unit‐level data. Because the survey was anonymous and did not collect identifiable institutional information, it was not possible to determine with certainty whether more than one respondent came from the same ICU or hospital. Therefore, the findings should not be interpreted as representing 128 distinct ICUs.

Questionnaires were excluded when they were substantially incomplete and/or did not meet the predefined eligibility criteria. Because the survey was designed to describe practice profiles across key domains, questionnaires lacking core eligibility information or most key practice variables were not retained. For the 128 questionnaires included in the final analysis, item‐level missing data were handled using available‐case analysis; therefore, denominators may vary across variables. The number of valid responses for each variable is reported in Section 3 and Table 1 where applicable. As the survey link was distributed through open professional networks and could be further shared by participants, the total number of individuals or ICUs reached could not be determined and a formal response rate could not be calculated.

**TABLE 1:** Reported CII management practices by healthcare setting.

Variable/category	Hospital trusts (*n* = 36)	University hospitals (*n* = 37)	Local healthcare organisations (*n* = 53)	Total valid responses, *n* (%)	Test; *p*
Standardised insulin infusion protocol					FFH exact; *p* = 0.555
Yes	35 (97.2)	33 (91.7)	49 (94.2)	117 (94.4)	
No	1 (2.8)	3 (8.3)	3 (5.8)	7 (5.6)	
Differentiated procedures for diabetes versus stress‐induced hyperglycaemia					FFH exact; *p* = 0.336
Yes	20 (57.1)	18 (50.0)	34 (65.4)	72 (58.5)	
No	15 (42.9)	18 (50.0)	18 (34.6)	51 (41.5)	
Insulin initiation threshold					FFH exact; *p* = 0.003
140 mg/dL	0 (0.0)	3 (8.1)	0 (0.0)	3 (2.4)	
160 mg/dL	8 (22.2)	3 (8.1)	7 (13.2)	18 (14.3)	
180 mg/dL	10 (27.8)	21 (56.8)	33 (62.3)	64 (50.8)	
200 mg/dL	18 (50.0)	10 (27.0)	13 (24.5)	41 (32.5)	
Glycaemic target range					FFH exact; *p* = 0.074
90–140 mg/dL	8 (22.2)	6 (16.2)	23 (43.4)	37 (29.4)	
100–160 mg/dL	12 (33.3)	10 (27.0)	9 (17.0)	31 (24.6)	
120–180 mg/dL	9 (25.0)	12 (32.4)	15 (28.3)	36 (28.6)	
140–200 mg/dL	7 (19.4)	9 (24.3)	6 (11.3)	22 (17.5)	
Glucose measurement method					FFH exact; *p* = 0.002
Arterial blood gas analyser	20 (55.6)	25 (69.4)	46 (86.8)	91 (72.8)	
Venous point‐of‐care strips	13 (36.1)	3 (8.3)	4 (7.5)	20 (16.0)	
Venous sample sent to central laboratory	1 (2.8)	4 (11.1)	1 (1.9)	6 (4.8)	
Real‐time sensor monitoring	2 (5.6)	2 (5.6)	1 (1.9)	5 (4.0)	
Arterial point‐of‐care strips	0 (0.0)	2 (5.6)	1 (1.9)	3 (2.4)	
Monitoring frequency before glycaemic stabilisation					FFH exact; *p* < 0.001
Every 15 min	0 (0.0)	3 (8.3)	0 (0.0)	3 (2.4)	
Every 60 min	14 (40.0)	14 (38.9)	12 (22.6)	40 (32.3)	
Every 2 h	15 (42.9)	9 (25.0)	8 (15.1)	32 (25.8)	
Every 4 h	5 (14.3)	6 (16.7)	31 (58.5)	42 (33.9)	
Every 6 h	0 (0.0)	2 (5.6)	2 (3.8)	4 (3.2)	
Real‐time sensor monitoring	1 (2.9)	2 (5.6)	0 (0.0)	3 (2.4)	
Monitoring frequency after glycaemic stabilisation					FFH exact; *p* = 0.834
Every 4 h	23 (65.7)	19 (51.4)	34 (64.2)	76 (60.8)	
Every 6 h	8 (22.9)	13 (35.1)	13 (24.5)	34 (27.2)	
Every 8 h	2 (5.7)	3 (8.1)	4 (7.5)	9 (7.2)	
Every 12 h	1 (2.9)	2 (5.4)	2 (3.8)	5 (4.0)	
Real‐time sensor monitoring	1 (2.9)	0 (0.0)	0 (0.0)	1 (0.8)	

*Note:* Data are presented as *n* (%), unless otherwise indicated. Column header sample sizes refer to respondents with available healthcare‐setting information. Percentages were calculated using valid responses for each variable; denominators may therefore vary because of item‐level missing data. Statistical tests and *p* values refer to exploratory comparisons across healthcare settings.

Abbreviations: CII, continuous intravenous insulin infusion; FFH, Fisher–Freeman–Halton exact test with Monte Carlo estimation.

### 2.3 Survey Instrument

The questionnaire was developed following a review of the available literature on CII management, glycaemic monitoring, insulin titration protocols and nursing care processes in ICU. The survey was informed by previously published instruments addressing nursing care processes and missed care components, but it was adapted to the specific context of insulin infusion management in critically ill patients.

Face and content validity were assessed through expert review by five clinicians with experience in critical care, ICU nursing practice and insulin infusion management, including intensivists and senior ICU nurses/nurse coordinators. Experts were asked to evaluate item clarity, clinical relevance, completeness and feasibility. Their feedback was incorporated through iterative revision of item wording and response options.

Before dissemination, the questionnaire was piloted among 10 ICU professionals who were not included in the final sample. The pilot phase assessed clarity, completion time, feasibility and clinical relevance of the survey items. Minor wording changes were made after pilot testing. No formal psychometric validation, content validity index, internal consistency assessment or test–retest reliability analysis was performed because the questionnaire was designed to describe reported practices rather than to measure a latent construct.

The questionnaire was administered in Italian. The manuscript was prepared in English after data analysis, and survey items and response categories were translated for reporting purposes by the authors and checked for consistency. The final questionnaire is provided as Supporting Information S1.

The final survey consisted of structured items covering the following domains:

- institutional characteristics
- availability of standardised insulin infusion protocols
- differentiated management for diabetes versus stress‐induced hyperglycaemia
- glycaemic target ranges
- insulin initiation thresholds
- glucose measurement methods
- monitoring frequency before glycaemic stabilisation
- monitoring frequency after stabilisation

### 2.4 Data Collection

The survey was administered electronically using a structured online questionnaire developed on Google Forms (Google LLC, Mountain View, CA, USA). Participation was voluntary and anonymous, and no identifiable personal or institutional data were collected. The survey link was distributed through professional networks and institutional contacts.

### 2.5 Variables

The primary organisational variable was the type of healthcare institution.

Clinical practice variables included:

- presence of a standardised insulin infusion protocol
- use of differentiated procedures for diabetes versus stress‐induced hyperglycaemia
- glycaemic target ranges
- insulin initiation thresholds
- glucose measurement methods
- monitoring frequency before stabilisation
- monitoring frequency after stabilisation

### 2.6 Statistical Analysis

Categorical variables are presented as frequencies and percentages. Continuous variables are reported as mean ± standard deviation (SD) and, where appropriate, as median and interquartile range (IQR). Percentages were calculated using valid responses for each variable.

Associations between healthcare setting and reported glycaemic management practices were explored using cross‐tabulations. Pearson's chi‐square test was planned when expected‐cell assumptions were satisfied. Because several contingency tables contained sparse cells, the Fisher–Freeman–Halton exact test with Monte Carlo estimation was used for the reported comparisons. Test names and *p* values are reported in Section 3 and Table 1. Given the exploratory design, *p* values were interpreted descriptively and no causal inference was made.

Statistical analyses were performed using IBM SPSS Statistics, version 30.

### 2.7 Ethics Statement

The study was approved by the Territorial Ethics Committee of the Calabria Region (approval no. 33, dated 20 January 2026). Participation was voluntary and anonymous. Completion of the questionnaire was considered implied informed consent.

## 3 Results

A total of 151 questionnaires were submitted. Of these, 23 were excluded because they were substantially incomplete and/or did not meet the predefined eligibility criteria, leaving 128 valid responses for the final analysis.

### 3.1 Institutional Characteristics and Protocol Availability

**FIGURE 1:**
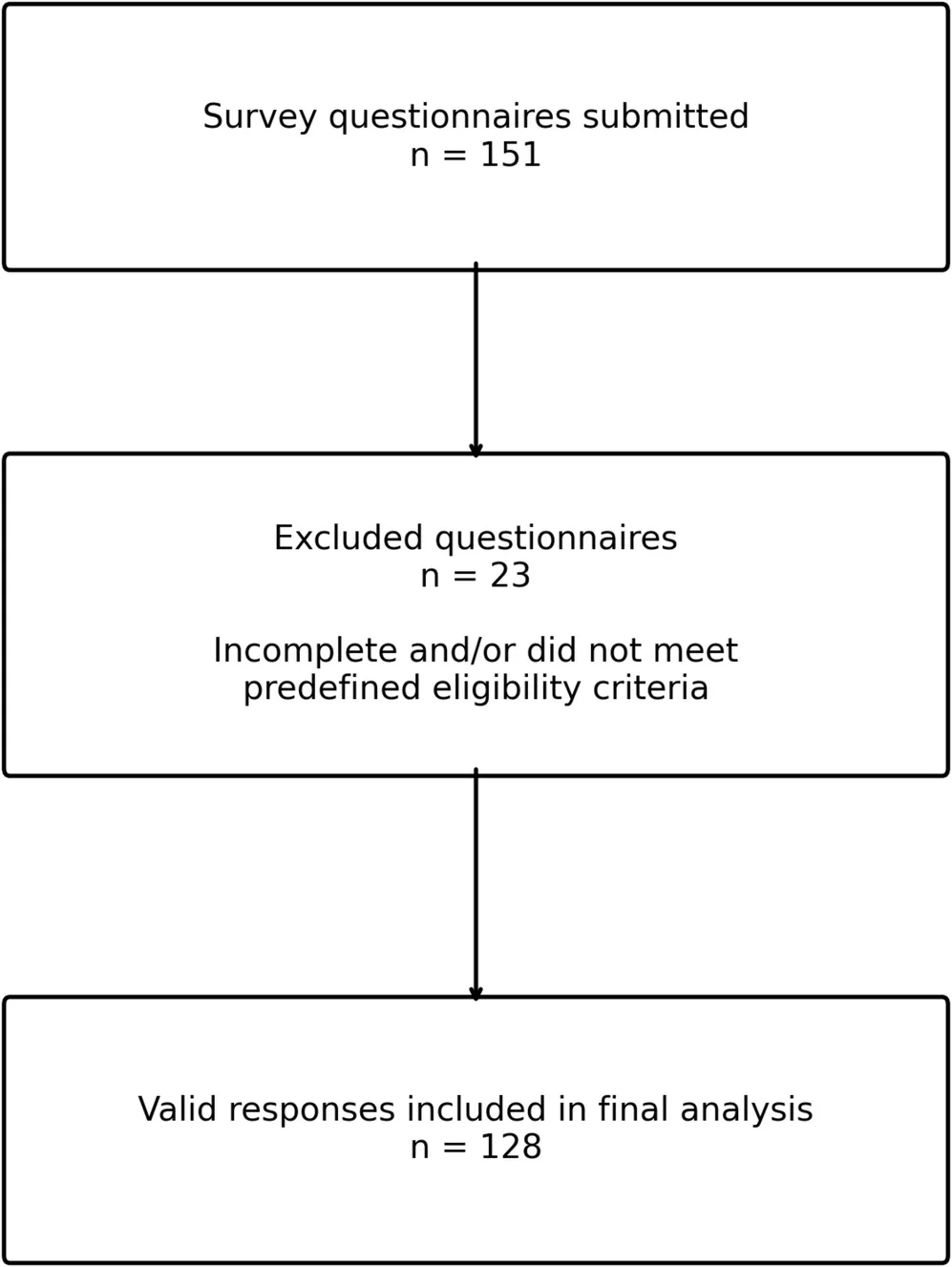
Survey response flow diagram. Eligible respondents were ICU nurses or nurse coordinators working in Italy and directly involved in, or familiar with, bedside management of continuous intravenous insulin infusion. Excluded questionnaires were substantially incomplete and/or did not meet the predefined eligibility criteria.

A total of 128 valid responses were included in the analysis. The flow of survey responses is shown in Figure 1. Overall, 151 questionnaires were submitted; 23 were excluded because they were substantially incomplete and/or did not meet the predefined eligibility criteria, leaving 128 valid responses for the final analysis. Healthcare setting was available for 126 respondents. These included hospital trusts (*n* = 36; 28.6%), university hospitals (*n* = 37; 29.4%) and local healthcare organisations (*n* = 53; 42.1%). Healthcare setting was missing for two respondents.

Standardised insulin infusion protocols were reported by 119 respondents (93.0%). Among respondents with both protocol and healthcare‐setting data available, 117 of 124 respondents (94.4%) reported a standardised insulin infusion protocol, as shown in Table 1. Overall, 74 respondents (57.8%) reported protocols including differentiated management strategies for patients with diabetes versus stress‐induced hyperglycaemia.

### 3.2 Glycaemic Targets and Insulin Initiation Thresholds

Considerable variability was observed in reported glycaemic target ranges. The most frequently reported targets were 90–140 mg/dL (*n* = 38; 29.7%), 120–180 mg/dL (*n* = 37; 28.9%) and 100–160 mg/dL (*n* = 31; 24.2%), while 140–200 mg/dL was reported by 22 respondents (17.2%).

Insulin initiation thresholds also varied across respondents. The most commonly reported threshold was 180 mg/dL (*n* = 64; 50.0%), followed by 200 mg/dL (*n* = 41; 32.0%), 160 mg/dL (*n* = 20; 15.6%) and 140 mg/dL (*n* = 3; 2.3%).

### 3.3 Monitoring Practices

The reported glucose‐monitoring interval before glycaemic stabilisation was based on respondents' estimates of usual practice rather than on audit data. Because real‐time sensor monitoring is not directly comparable with intermittent glucose checks, monitoring frequency is reported primarily as categorical data. When real‐time sensor monitoring was coded as 0 min for descriptive purposes, the mean reported monitoring interval before stabilisation was 142.7 ± 89.2 min, with a median of 120 min and an IQR of 60–240 min. In a sensitivity analysis excluding respondents reporting real‐time sensor monitoring before stabilisation, the mean interval remained above 2 h, at 147.1 ± 87.7 min, with a median of 120 min and an IQR of 60–240 min. After stabilisation, the mean reported monitoring interval was 5.15 ± 1.93 h, with a median of 4 h and an IQR of 4–6 h.

Before glycaemic stabilisation, 40 respondents (32.3%) reported hourly monitoring, whereas 32 (25.8%) reported monitoring every 2 h, 42 (33.9%) every 4 h and 4 (3.2%) every 6 h. Real‐time sensor monitoring before stabilisation was reported by 3 respondents (2.4%). After stabilisation, most respondents reported monitoring every 4 h (*n* = 76; 60.8%) or every 6 h (*n* = 34; 27.2%).

Respondents reported a mean of 2.96 ± 1.74 patients receiving continuous intravenous insulin infusion in their ICU during the previous week.

### 3.4 Exploratory Comparisons by Healthcare Setting

Exploratory comparisons between healthcare setting and reported glycaemic management practices are summarised in Table 1.

No clear differences were observed across healthcare settings for standardised insulin infusion protocol availability (Fisher–Freeman–Halton exact test, *p* = 0.555), differentiated procedures for diabetes versus stress‐induced hyperglycaemia (Fisher–Freeman–Halton exact test, *p* = 0.336), glycaemic target ranges (Fisher–Freeman–Halton exact test, *p* = 0.074) or monitoring frequency after stabilisation (Fisher–Freeman–Halton exact test, *p* = 0.834).

Exploratory comparisons suggested differences across healthcare settings for insulin initiation thresholds (Fisher–Freeman–Halton exact test, *p* = 0.003), glucose measurement methods (Fisher–Freeman–Halton exact test, *p* = 0.002) and monitoring frequency before glycaemic stabilisation (Fisher–Freeman–Halton exact test, *p* < 0.001).

## 4 Discussion

This national cross‐sectional survey found substantial variability in self‐reported CII management practices among ICU nurses and nurse coordinators, despite the high reported availability of standardised insulin infusion protocols. The main areas of variability concerned insulin initiation thresholds, glycaemic target ranges, glucose measurement methods and monitoring frequency before glycaemic stabilisation. These findings suggest a potential gap between protocol availability, guideline recommendations and reported routine practice. However, because adherence to local protocols was not directly audited, the results should be interpreted as describing reported practice variability rather than objectively measured protocol implementation.

Healthcare setting was used as an exploratory organisational variable to describe whether reported CII practices differed across different institutional contexts. These comparisons should not be interpreted causally, particularly because the survey did not collect detailed information on staffing models, local governance, ICU case mix or protocol implementation processes. Nevertheless, the descriptive patterns observed in Table 1 suggest that some practices varied by setting. For example, hospital trusts more frequently reported an insulin initiation threshold of 200 mg/dL, whereas university hospitals and local healthcare organisations more frequently reported 180 mg/dL. Local healthcare organisations more frequently reported arterial blood gas analyser use for glucose measurement and less frequent monitoring before glycaemic stabilisation. These differences may reflect local protocols, available resources or workflow organisation, but they require confirmation in future studies specifically designed to evaluate institutional determinants of CII practice.

The observed variability should be interpreted in the context of current guideline recommendations for glycaemic control in critically ill adults [13]. In this study, the most frequently reported insulin initiation threshold was 180 mg/dL, which is consistent with recommendations that persistent hyperglycaemia ≥ 180 mg/dL should trigger glycaemic management protocols [13]. However, reported glycaemic targets were heterogeneous. Although some respondents reported conventional or moderate ranges such as 120–180 mg/dL or 140–200 mg/dL, a substantial proportion reported lower targets, including 90–140 mg/dL. Although the reported range of 90–140 mg/dL is not identical to the intensive target range of 80–139 mg/dL addressed in current recommendations, it remains lower than the more conventional ranges generally suggested for critically ill adults. This finding therefore suggests that some respondents reported relatively tight glycaemic targets, highlighting variability in how guideline recommendations are interpreted and translated into reported practice [13].

Monitoring frequency before glycaemic stabilisation was one of the most clinically relevant areas of variability. In the present survey, respondents reported a mean monitoring interval before stabilisation of more than 2 h, whereas current guidelines recommend frequent glucose monitoring, generally at intervals of 1 h or less, during periods of glycaemic instability [13]. Because monitoring intervals were based on respondents' estimates of usual practice rather than audit data, these findings should be interpreted cautiously. Nevertheless, they are consistent with a pilot study conducted in the United States showing that adherence to hourly glucose monitoring during insulin infusion can be difficult to achieve in ICU practice [14]. A separate United States intensive care unit study found that delayed glucose reassessment was associated with an increased risk of hypoglycaemia, supporting the clinical relevance of timely monitoring during CII therapy [15].

Several factors may plausibly contribute to the observed gap between guideline recommendations and reported routine practice. CII management is inherently labour‐intensive, requiring repeated glucose measurements, timely insulin dose adjustments, documentation and escalation when glucose values are outside target ranges [5, 6]. These activities are largely nurse‐led at the bedside and must be integrated into broader ICU workflows. Although nursing workload, staffing levels, skill mix, protocol familiarity and training were not directly measured in this study, they represent plausible implementation‐related factors that may help explain why recommended monitoring intervals are difficult to maintain in routine practice [5, 6, 16]. Therefore, these findings should not be interpreted as demonstrating a direct effect of workload or staffing on CII management, but rather as identifying areas where future studies should directly evaluate organisational and nursing‐related barriers to protocol adherence.

Continuous glucose monitoring and closed‐loop insulin delivery systems have been investigated in studies including cardiovascular intensive care research in the United States and closed‐loop system development in Japan as potential tools to support glycaemic management in critically ill patients [8, 9, 10, 11, 12]. However, evidence supporting their routine use in ICU settings remains limited, and issues related to accuracy, safety, integration with insulin titration protocols, staff training, costs and local feasibility require further evaluation. In the present survey, CGM use was reported by only a small minority of respondents; therefore, our findings only describe its limited reported use and do not allow assessment of how CGM is integrated into routine ICU workflows. Future studies should assess whether CGM can safely reduce the burden of repeated bedside glucose testing and improve the timeliness of insulin titration in selected ICU populations.

Overall, these findings suggest variability between protocol availability, guideline recommendations and self‐reported routine practice. They also identify monitoring frequency before glycaemic stabilisation as a clinically relevant area for future quality improvement and implementation research in critical care nursing.

### 4.1 Strengths and Limitations

This study provides national survey data on self‐reported glycaemic management practices among ICU nurses and nurse coordinators, with a specific focus on continuous intravenous insulin infusion management. Several limitations should be acknowledged. First, responses were self‐reported and may not fully reflect actual clinical practice or adherence to local protocols. Second, the use of convenience and snowball sampling may have introduced selection bias and may limit generalisability. Third, the unit of analysis was the individual respondent, and the anonymous survey design did not allow us to verify whether multiple responses originated from the same ICU or hospital. Therefore, the results should be interpreted as self‐reported practices of ICU nurses and nurse coordinators rather than as definitive unit‐level estimates. Fourth, detailed respondent characteristics, such as professional role, years of ICU experience, ICU specialty and nurse‐to‐patient ratios, were not collected; this limited our ability to explore whether individual professional experience or ICU type influenced reported CII management practices. Fifth, although the questionnaire underwent expert review by five clinicians and pilot testing among 10 ICU professionals, no formal psychometric validation, content validity index, internal consistency assessment or test–retest reliability analysis was performed. Therefore, the survey should be interpreted as an exploratory instrument designed to describe reported practices. Sixth, no patient‐level clinical outcomes were available; therefore, the direct effect of the observed variability on hypoglycaemia, hyperglycaemia, morbidity or mortality could not be assessed. Finally, all comparisons according to healthcare setting were exploratory, and the presence of sparse cells in some contingency tables may have limited the robustness of inferential analyses. Findings should therefore be interpreted with appropriate caution.

### 4.2 Generative AI Use Statement

During revision of this manuscript, the authors used OpenAI ChatGPT (GPT‐5.6 Thinking; accessed July 2026) solely to support English‐language editing and the organisation of responses to editorial comments. No generative AI tool was used to generate or analyse study data. All output was critically reviewed, verified and edited by the authors, who take full responsibility for the final content.

### 4.3 Implications for Clinical Practice and Further Research

The findings suggest that intensive care unit nurses and nurse coordinators report variable practices in continuous intravenous insulin infusion management, particularly in relation to initiation thresholds, glucose measurement methods and monitoring frequency before glycaemic stabilisation. Clinical services should review local insulin infusion protocols, specify monitoring intervals during glycaemic instability and ensure clear escalation procedures when glucose checks are delayed.

Actionable strategies include targeted staff education, periodic audit and feedback, electronic reminders or bedside prompts and multidisciplinary review of insulin infusion workflows. Future multicentre studies should use audited unit‐level and patient‐level data, account for clustering of respondents within intensive care units and directly evaluate staffing, workload, skill mix, protocol adherence, hypoglycaemia and other clinical outcomes. Prospective implementation studies should also assess whether workflow redesign or selected glucose‐monitoring technologies improve the timeliness and safety of insulin titration.

## 5 Conclusions

Despite the widespread reported availability of standardised insulin infusion protocols, substantial variability was observed in self‐reported CII management practices among ICU nurses and nurse coordinators. Variability was particularly evident for glycaemic targets, insulin initiation thresholds, glucose measurement methods and monitoring frequency before glycaemic stabilisation.

The reported monitoring interval before stabilisation frequently exceeded the frequency recommended during periods of glycaemic instability, suggesting a potential implementation gap in routine ICU practice. However, because this study was based on self‐reported data and did not directly assess adherence to local protocols, staffing levels, nursing workload or patient outcomes, these findings should be interpreted with caution.

Reducing variability in CII management may require not only evidence‐based protocols, but also structured implementation strategies, staff education, audit and feedback and organisational support. Future research should directly evaluate the nursing and organisational barriers that may affect timely glucose monitoring and safe insulin titration in critically ill patients.

## Funding

The authors have nothing to report.

## Ethics Statement

The study was approved by the Territorial Ethics Committee of the Calabria Region (approval no. 33, dated 20‐01‐2026).

## Consent

Participation was voluntary and anonymous. Completion of the questionnaire was considered implied informed consent.

## Conflicts of Interest

The authors declare no conflicts of interest.

## Supporting information


**Supporting Information S1:** Survey questionnaire.

## Data Availability

The anonymised data supporting the findings of this study are available from the corresponding author upon reasonable request, subject to applicable ethical and institutional requirements.
