# Supplementary material for: Variability in Intravenous Insulin Infusion Management in Italian Intensive Care Units: A National Cross‐Sectional Survey of Reported Nursing Practice
Source: Nurs Crit Care. 2026 Aug 31;31(5):e70656. doi: 10.1111/nicc.70656 (PMC13527653; doi:10.1111/nicc.70656)
Supplement: Supplementary file 1 [file NICC-31-0-s001.docx]

**Supplementary File 1. Survey questionnaire**

*Questionnaire administered to ICU nurses and nurse coordinators*

**Note.** The questionnaire was administered in Italian through Google Forms. The original Italian version is provided below, followed by an English translation for transparency and reproducibility.

# Original Italian questionnaire

| **No.** | **Question** | **Response options / format** |
| --- | --- | --- |
| 1 | In quale UTI lavori? | Azienda Ospedaliera Azienda Ospedaliero Universitaria Azienda sanitaria provinciale |
| 2 | Numero posti letto | Risposta numerica |
| 3 | Numero di pazienti in terapia infusionale continua di insulina (ultima settimana) | Risposta numerica |
| 4 | Adozione di protocollo standard | Sì No |
| 5 | Adozione di procedure differenziate per diabete o iperglicemia da stress | Sì No |
| 6 | Inizio terapia in caso di glicemia superiore a | 140 mg/dL 160 mg/dL 180 mg/dL 200 mg/dL |
| 7 | Target terapeutico glicemia | 140–200 mg/dL 120–180 mg/dL 100–160 mg/dL 90–140 mg/dL |
| 8 | Dosaggio glicemia con | Striscette su sangue arterioso Striscette su sangue venoso Sangue venoso inviato a laboratorio analisi Emogasanalisi Monitoraggio in tempo reale con sensori |
| 9 | Dosaggio glicemia (prima della stabilizzazione) ogni | In tempo reale con sensori 15 minuti 60 minuti 2 ore 4 ore 6 ore |
| 10 | Dosaggio glicemia (dopo la stabilizzazione) ogni | In tempo reale con sensori 4 ore 6 ore 8 ore 12 ore |

# English translation

| **No.** | **Question** | **Response options / format** |
| --- | --- | --- |
| 1 | In which ICU do you work? | Hospital trust University hospital Local healthcare organisation |
| 2 | Number of ICU beds | Numerical response |
| 3 | Number of patients receiving continuous intravenous insulin infusion during the previous week | Numerical response |
| 4 | Adoption of a standard protocol | Yes No |
| 5 | Adoption of differentiated procedures for diabetes or stress-induced hyperglycaemia | Yes No |
| 6 | Insulin therapy initiated when blood glucose is above | 140 mg/dL 160 mg/dL 180 mg/dL 200 mg/dL |
| 7 | Therapeutic blood glucose target | 140–200 mg/dL 120–180 mg/dL 100–160 mg/dL 90–140 mg/dL |
| 8 | Blood glucose measurement method | Arterial point-of-care strips Venous point-of-care strips Venous sample sent to central laboratory Blood gas analyser Real-time sensor monitoring |
| 9 | Blood glucose measurement frequency before glycaemic stabilisation | Real-time sensor monitoring Every 15 minutes Every 60 minutes Every 2 hours Every 4 hours Every 6 hours |
| 10 | Blood glucose measurement frequency after glycaemic stabilisation | Real-time sensor monitoring Every 4 hours Every 6 hours Every 8 hours Every 12 hours |
